# Physico-chemical composition and sensory analysis of female broiler breast meat of local Anadolu-T and commercial genotypes slaughtered at different ages

**DOI:** 10.5194/aab-69-653-2026

**Published:** 2026-11-10

**Authors:** Erdem Çağlar Küpeli, Metin Petek, Zehra Avci Küpeli, Eyüphan Petek

**Affiliations:** 1 Department of Animal Science, Faculty of Veterinary Medicine, Bursa Uludag University, 16059 Nilüfer-Bursa, Türkiye; 2 Department of Pathology, Faculty of Veterinary Medicine, Near East University, Nicosia/TRNC, Mersin 10, Türkiye; 3 Department of Obstetrics and Gynecology, Faculty of Veterinary Medicine, Bursa Uludag University, 16059 Nilüfer-Bursa, Türkiye

## Abstract

This study was made to compare the physico-chemical meat quality properties, and sensory attributes of broiler meat of local Anadolu-T and commercial genotypes slaughtered at different ages. A total of 150 d-old female chicks were randomly assigned to three groups with five replicates each. The breast meat samples from each genotype were collected randomly after slaughter at 37 and 44 d of age. Breast meat proximal composition of pectoralis major was determined, as well as the sensory analysis using a 9-point hedonic scale. The effects of slaughter age were found to be significant for meat pH ( P=0.003 ), lightness ( P=0.001 ), redness ( P=0.018 ), yellowness ( P<0.001 ) and chrome ( P<0.001 ) color characteristics. Shear force values increased significantly with the slaughter age ( P<0.001 ). The breast meat of Cobb 500 had significantly the greatest meat pH ( P<0.01 ), yellow/blue coordinate ( P=0.015 ) and color intensity-chroma ( P=0.003 ) values, whereas the breast meat of Anadolu-T had significantly the greatest lightness ( P=0.018 ), and the lowest yellow/blue coordinate ( P=0.015 ) and color intensity-chrome values ( P=0.015 ). Slaughter age and genotype significantly affected the moisture ( P<0.001 ) and protein level ( P=0.002 ) of meat. Breast meat samples of the Ross 308 genotype had a significantly greater cooking loss value ( P=0.007 ). There were no significant differences for all sensory characteristics among the samples obtained from all genotypes. In conclusion it can be said that the genotype and slaughter age play crucial roles in determining the proximate composition, meat color and sensory characteristics of broiler breast meat. The local Turkish genotype, *Anadolu-T*, seems to exhibit competitive meat quality compared to commercial broiler strains.

## 1 Introduction

Numerous determinants such as strain, slaughter age and husbandry practices play significant roles in defining the physical and chemical attributes of broiler meat, including tenderness and flavor (Mir et al., 2017, Özbek et al., 2020; Hailemariam et al., 2022; Yue et al., 2024; Selvi et al., 2026). Poultry gender has a greater influence on muscle myopathies, water-holding capacity and shear properties (Li et al., 2019; Siddique et al., 2024). Males tend to have thicker and longer fillets, a higher incidence of white striping and greater cooking losses compared to females (Maynard et al., 2023). Broiler slaughter age influences muscle development, tissue characteristics and fat deposition; overall, this affects the physical, chemical and sensory characteristics of meat (Mir et al., 2017, Suliman et al., 2026). The protein content of breast meat increases with advancing slaughter age (Park et al., 2021). while slaughter age and strain affect water-holding capacity and cooking loss of meat (Mueller et al., 2023).

Ross 308 and Cobb 500 are the main commercial broiler genotypes raised for broiler meat production in Türkiye, and these birds account for almost all parts of flocks. The use of local poultry in egg and broiler production, especially in extensive systems, is presented as an alternative practice to conventional farming (Meira et al., 2022), and it is getting popular for a sustainable production. As in breast muscle myopathies of local and commercial-meat-type chickens (Avcı-Küpeli et al., 2025), there might be significant differences in terms of their meat quality. Broiler genotype strongly affected the physico-chemical and sensory characteristics of broiler meat, primarily through differences in muscle fiber composition and ultimate pH (Hailemariam et al., 2022; Özbek et al., 2020). Commercial broiler genotypes generally produce lighter breast meat, whereas local genotypes present lower lightness values, and higher redness or yellowness characteristics (Samakradhamrongthai et al., 2026). Since 2000, a local broiler chicken, called Anadolu-T, was put into commercial broiler meat production as a fast-growing broiler in Türkiye (Tagem Arge and Innovasyon, 2020). The first findings about the overall performance of the Anadolu-T genotype were found to be competitive but lower than commercial broilers (Erensoy and Sarıca, 2023; Güler and Petek, 2026). There were no published studies on nutritive values and sensory characteristics of fresh meat of Anadolu-T chickens compared to commercial strains. Understanding the sensory characteristics of female meat raised especially in a slatted-floor housing system could provide key insights into improving meat quality and consumer satisfaction. Depending on welfare legislation and regional practices, slatted-floor housing systems are being examined in some parts of the world as an alternative to conventional deep litter and cage housing systems (Topal and Petek, 2021; Aslan et al., 2024). In addition, the practice of raising males and females separately is becoming increasingly common worldwide, optimizing broiler meat production (England et al., 2023; Ebrahimi et al., 2026). It would be valuable to identify the best genotype for achieving optimal meat quality of the female broiler by comparing a local Turkish broiler genotype with two commonly used commercial broiler genotypes. This study was made to investigate the physico-chemical quality composition and sensory analysis of female broiler meat of local Anadolu-T and two commercial genotypes (RossPM3 and Cobb 500) slaughtered at different ages.

## 2 Material and methods

The study was approved by the Bursa Uludag University of Animal Experiment Ethic Committee for Scientific Research (Bursa Uludag University 2022, 02/01). The breast meat samples were collected from three different genetic strains slaughtered at 37 and 44 d ( 3×2 ).

### 2.1 Animals, housing, management and slaughter

A total of 150 fast-growing (*Ross 308, Cobb 500* and *local Anadolu-T*) female broiler chicks were raised in experimental pens until 44 d of age (DOA). There were five replicates (15 experimental pens in total) in each group in the experiment, with 10 broilers in each replicate. The experimental groups were housed in similar environmental conditions for floor space (1 $m^{2}$ for each replicate), stocking density, and drinker or feeder space in slatted floors until the end of the experiment (Aviagen-ROSS Broiler Pocket Guide, 2025).

The surface of the slats was covered by chicken paper (100 % biodegradable paper) during the first week of the experiment. Continuous lighting (daylight and artificial light in a day) was used in the first week of the experiment. After that, 6 h of dark and 18 h of light was applied until 3 d before slaughter. The broiler chicks in the groups were fed with a multiphase corn and soymeal-based diets (*starter*: 23 % crude protein and 2950 $\mathrm{kcal}\;{\mathrm{kg}}^{-1}$ ME, for days 0–15; *grower I*: 21 % crude protein and 3000 $\mathrm{kcal}\;{\mathrm{kg}}^{-1}$ ME for days 15–30; *grower II*: 20 % crude protein and 3050 $\mathrm{kcal}\;{\mathrm{kg}}^{-1}$ ME for days 30–37; and *finisher*: 19 % crude protein and 3100 $\mathrm{kcal}\;{\mathrm{kg}}^{-1}$ ME, for days 37–44), which are produced by a commercial feed company. Broilers were fed a crumble-form diet during the first 15 d and then a pelleted diet until the end of the experiment. Feed and water were provided ad libitum throughout the experimental period.

The broilers were slaughtered at 37 and 44 d of age in standard conditions for poultry (HSA, 2013). After cutting, bleeding, scalding and defeathering of the carcasses, the whole carcasses were cooled for 3 h at 4 °C. The carcass weights of Ross 308, Anadolu-T and Cobb 500 were 1574, 1429 and 1726 g on day 37; and 2162, 2043 and 2140 g on day 44, respectively. Meanwhile, breast meat weights were 636, 579 and 674 g on day 37; and 1020, 934 and 985 g on day 44, respectively. In the study, five carcasses at 37 DOA and 10 carcasses at 44 DOA were selected to analysis, and the whole breast meat and legs were removed from the randomly selected carcasses (Löhren, 2012, Turkish Official Gazette, 2011; Barbut, 2016). Breast meat samples from the pectoralis major muscle and thigh meat samples from the legs were collected to perform analysis. The meat samples were placed in a plastic bag, covered with ice pieces and sent to the laboratory after the chilling process. The meat samples were kept in the refrigerator during the analysis.

### 2.2 Data collection

All experiments – except for the basal proximate composition of protein, fat and humidity content of the samples – were performed as part of the meat and carcass evaluation at the laboratory of the Faculty of Veterinary Medicine of Bursa Uludag University. Meat color traits of the samples were measured with a colorimeter (PCE-XXM 20, PCE Instruments LTD) according to the CIE $L^{*}a^{*}b^{*}$ system (D65/10 °) on the cranial surface of breast muscle (Keskin et al., 2017) after calibration using a specific white board. The lightness ( $L^{*}$ ) values ranged from black (0) to white (100), while the red/green coordinate ( $a^{*}$ ) and yellow/blue coordinate ( $b^{*}$ ) both ranged from red or blue ( -60 ) to blue or yellow ( +60 ) (Kralik et al., 2018). The chroma value (color intensity/saturation index; $C^{*}=(a^{*2}+b^{*2}{)}^{1/2}$ ), hue angle as arctan values ( $h^{\circ }={\mathrm{tan}}^{-1}(b^{*}/a^{*})\cdot 180/\pi$ ) and total difference ( $\Delta E=(L^{2}+a^{2}+b^{2})1/2$ ) were calculated from the values $a^{*}$ and $b^{*}$ for each meat sample (King et al., 2023).

The shear force (SF) of meat was evaluated using cooked samples (cooking in a water bath until reaching an internal temperature of 82–85 °C). After cooking, the samples were cooled to room temperature and stored at 4 °C for 24 h (Wheeler et al., 1997). Approximately 3–5 ${\mathrm{cm}}^{3}$ of the sample taken from the middle of the breast meat was weighed before and after cooking. Shear force was measured using texture analyzer (Zwick/Roell 20.05, Germany), using the Warner–Bratzler cutting blade head to measure the peak shear force, total shear energy (cutting work) and the number of shear peaks (Zhuang and Savage, 2013). The pH analysis was carried out at three different points of breast muscle using a pH meter (Hanna HI99163).

Crude protein, fat and moisture content of the meat samples were analyzed in a private food analysis laboratory using the AOAC Analytical Methods (Thiex, 2023). Crude protein determination in meat samples was carried out in three stages with the Kjeldahl method as digestion neutralization and titration with the distillation stage (method 992.15). Crude fat analysis in meat samples was made according to the Soxhlet method (method 960.39). Cooking loss values of meat samples was determined as reported by Kondaiah et al. (1985). Drip loss of breast meat samples was measured using a plastic bag drip method according to the procedure (Honikel, 1998).

The samples for sensory analysis were prepared and divided into equal-sized pieces ( 1cm×1cm×1cm ). Then all samples were placed on trays, covered with aluminum foil and then cooked at 20 °C for 45 min. Analyzes were carried out with panelists who were trained on the subject, had been in at least one sensory analysis panel before and who did not know which chicken they were eating. Participants were allowed to drink water to avoid any taste in their mouths while evaluating between groups. Panelists were asked to evaluate from 1 to 9 for the sensory traits of the chicken meats (Table 1). In the arrangement of the panel, a 9-point hedonic scale reported by Wichchukit and O'Mahony (2015) was used. In the scoring, 1 is the lowest (dislike extremely), 9 means the highest value (Like/good, extremely like) and 5 is neither like nor dislike (control) (Cox, 2013, Tura et al., 2024).

**Table 1:** Description of a 9-point hedonic scale and parameter using for sensory analysis of breast meat.

Category	1/Dislike	5/Control	9/Like
Color	Whitish	Neither … nor …	Yellowish
Tenderness/toughness (felt between two fingers)	Very soft	Neither … nor …	Extremely hard
Odor intensity	Not perceived	Neither … nor …	Extremely strong
Moisture (juiciness perceived during chewing)	1	Neither … nor …	9
Flavor/taste	Not perceived (extremely undesirable)	Neither … nor …	Extremely good/desirable
Texture (perception of fiber left in the mouth during chewing)	1	Neither … nor …	9
Aftertaste (overall impression and desire to eat again)	Not good (extremely undesirable)	Neither … nor …	Extremely good/desirable

Note: 1=the lowest (dislike extremely); 5=neither like nor dislike (control); 9=the highest (like/good, extremely like).

### 2.3 Statistical analysis

The results of the study are presented as means and standard error of means. A two-way ANOVA test was used for statistical analysis for the physico-chemical traits of breast meat using SPSS^®^ Computer Software 28.00 (IBM Corp 2021), while that of sensory characteristics was analyzed using a nonparametric Kruskal–Wallis test. When differences between groups after ANOVA were found to be significant, the Duncan's multiple range test was used for comparison (Snedecor and Cochran, 1989; Starbuck, 2023). The generalized linear model (GLM) is described as follows:

1 Yijk=μ+Ai+Bj+A×B+eijk.

A : age; B : genotype; A×B : an interaction; i : 1,2 (1: 37 DOA, 2: 44 DOA), j : 1,2,3 (1: Ross 308, 2: Anadolu-T, 3: Cobb 500), μ , a constant; e , an error term.

## 3 Results

Warner–Bratzler (WB) peak shear force values of breast meat in different genotypes of broilers are presented in Table 2. While slaughter age had a significant effect on peak shear force and shear energy ( p<0.001 , P=0.009 ), the effect of genotype on peak shear force, number of peaks and shear energy was found to be not significant. Breast meat samples taken from broilers slaughtered at 44 d of age (DOA) showed significantly higher peak shear force and shear energy values compared to those slaughtered at 37 DOA.

**Table 2:** Shear force values of breast meat in different broiler genotypes slaughtered at different ages (mean ± SEM).

Groups	Peak shear force,	Number of peaks	Shear energy,
	N		Nmm , J
Age			
37 DOA	20.93±1.48	12.73±0.99	108.15±10.18
44 DOA	27.93±1.05	15.04±0.70	142.51±7.25
Genotype			
Ross 308	24.69±1.58	14.95±1.05	136.44±10.81
Anadolu-T	24.03±1.59	13.77±1.06	119.26±10.82
Cobb 500	24.56±1.59	12.93±1.05	120.28±10.80
Age × genotype			
37 × Ross 308	21.37±2.57	12.89±1.71	122.08±17.65
37 × Anadolu-T	19.74±2.56	13.52±1.72	105.05±17.60
37 × Cobb 500	21.68±2.56	11.79±1.70	97.33±17.30
44 × Ross 308	28.01±1.82	17.02±1.21	150.81±12.45
44 × Anadolu-T	28.32±1.81	14.02±1.20	133.47±12.40
44 × Cobb 500	27.45±1.80	14.07±1.19	143.23±12.41
P value			
Age	0.001	0.066	0.009
Genotype	0.852	0.404	0.458
Age × genotype	0.914	0.484	0.808

DOA: days of age.

Breast color characteristics of female broilers at different slaughter ages of different broiler genotypes are presented in Table 3.

**Table 3:** Age and genotype effects on color characteristics of broiler breast meat (mean ± SEM).

Groups	Lightness, $L^{*}$	Red/green	Yellow/blue	Hue angle, h°	Color	Total color
		coordinate, $a^{*}$	coordinate, $b^{*}$		Intensity-chrome, $C^{*}$	difference, $\Delta E^{*}$
Age						
37 DOA	46.28±1.20	3.81±2.61	14.38±1.40	14.20±14.46	18.86±1.60	1315.87±49
44 DOA	52.11±0.85	-4.05±1.84	3.84±0.89	8.63±10.22	9.06±1.13	1418.05±34
Genotype						
Ross 308	$49.62\pm 1.23^{\text{a,b}}$	0.98±2.77	$10.01\pm 1.49^{b}$	21.69±15.36	$16.04\pm 1.69^{a}$	1430.77±52
Anadolu-T	$51.65\pm 1.26^{a}$	-0.45±2.69	$5.52\pm 1.47^{a}$	24.66±15.32	$8.89\pm 1.70^{b}$	1394.95±52
Cobb 500	$46.32\pm 1.27^{b}$	-0.90±2.70	$11.80\pm 1.46^{b}$	-12.10±15.33	$16.94\pm 1.69^{a}$	1275.14±52
Age × genotype						
37 × Ross 308	46.22±2.07	7.88±4.32	17.02±2.43	34.65±25.16	23.46±2.74	1400.70±85
37 × Anadolu-T	50.53±2.09	2.74±4.41	8.87±2.44	28.86±25.14	10.31±2.77	1354.43±85
37 × Cobb 500	42.09±2.08	0.81±4.52	17.24±2.42	-20.91±25.04	22.80±2.76	1192.48±84
44 × Ross 308	53.01±1.26	-5.91±3.21	2.99±1.72	8.72±17.73	8.63±1.97	1460.83±60
44 × Anadolu-T	52.77±1.47	-3.63±3.31	2.17±1.73	20.46±17.72	7.47±1.96	1435.48±60
44 × Cobb 500	50.55±1.27	-2.61±3.20	6.36±1.73	-3.30±17.71	11.08±1.95	1357.81±59
P value						
Age	0.001	0.018	0.001	0.755	0.001	0.095
Genotype	0.018	0.882	0.015	0.183	0.003	0.096
Age × genotype	0.215	0.402	0.230	0.604	0.045	0.749

DOA: days of age; a, b: values with different superscripts in the same columns differ significantly from each other.

Slaughter age significantly affected the lightness ( $L^{*}$ ), redness color coordinate ( $a^{*}$ ), yellow color coordinate ( $a^{*}$ ) and color intensity (chroma) properties of breast meat ( P<0.001 , P=0.018 , P<0.001 ). Lightness ( $L^{*}$ ), yellowness color coordinate ( $b^{*}$ ) and color intensity values of breast meat samples were significantly affected by genotype differences ( P=0.018 , P=0.015 and P=0.003 ). Interactions between genotype × slaughter age for color intensity were found to be significantly important ( P=0.045 ).

Proximate composition and physico-chemical analysis of breast meat in different genotypes of broilers are presented in Table 4.

**Table 4:** Proximate composition and physico-chemical analysis of breast meat of different broiler genotypes (mean ± SEM).

Groups	pH	Moisture, %	Protein, %	Crude fat, %	Cooking loss, %	Drip loss, %
Age						
37 DOA	5.82±0.03	73.89±0.22	23.12±0.22	1.80±0.14	23.06±0.831	5.07±0.942
44 DOA	5.92±0.02	75.05±0.15	22.25±0.16	1.70±0.11	22.93±0.587	7.17±0.666
Genotype						
Ross 308	$5.83\pm 0.03^{a}$	$75.35\pm 0.23^{a}$	$22.34\pm 0.232^{a}$	1.60±0.16	$25.16\pm 0.881^{a}$	5.30±0.999
Anadolu-T	$5.84\pm 0.03^{a}$	$75.20\pm 0.76^{a}$	$21.91\pm 0.231^{a}$	1.72±0.15	$20.96\pm 0.880^{b}$	6.52±0.998
Cobb 500	$5.94\pm 0.02^{b}$	$72.92\pm 0.83^{b}$	$23.81\pm 0.233^{b}$	1.92±0.16	$22.85\pm 0.881^{b}$	6.55±0.998
Age × genotype						
37 × Ross 308	5.72±0.04	75.45±0.37	22.49±0.379	1.23±0.26	27.59±1.439	5.54±1.63
37 × Anadolu-T	5.78±0.04	74.75±0.36	22.13±0.378	2.17±0.25	17.19±1.378	4.18±1.58
37 × Cobb 500	5.96±0.04	71.50±0.35	24.75±0.377	2.00±0.24	24.39±1.437	5.50±1.64
44 × Ross 308	5.93±0.03	75.14±0.26	22.19±0.268	1.98±0.18	22.74±1.468	5.06±1.65
44 × Anadolu-T	5.90±0.03	75.66±0.25	21.68±0.268	1.27±0.17	24.73±1.468	8.86±1.62
44 × Cobb 500	5.93±0.04	74.35±0.27	22.88±0.269	1.84±0.18	21.32±1.469	7.59±1.63
P value						
Age	0.003	0.001	0.002	0.586	0.901	0.076
Genotype	0.010	0.001	0.001	0.361	0.007	0.607
Age × genotype	0.011	0.001	0.038	0.002	0.001	0.201

DOA: days of age; a, b: values with different superscripts in the same columns differ significantly from each other.

Moisture, pH and crude protein content of breast meat were significantly affected by slaughter age ( P<0.001 , P=0.003 , P=0.002 ). There were no significant differences in cooking loss values of breast meat collected at 37 and 44 DOA. Breast meat moisture was found to be significantly lowest in the Cobb 500 genotype ( P<0.001 , P<0.01 ), while protein content and acidity level was significantly highest in the Cobb 500 genotype ( P=0.007 ) and cooking loss was significantly lowest in the Anatolian-T genotype ( P=0.007 ). Age–genotype interaction was found to be significant in terms of breast meat moisture, pH, crude protein, crude fat and cooking loss.

Sensory characteristics of breast and thigh meat obtained from different broiler genotypes are presented in Table 5. There were no significant differences for all sensory quality characteristics of breast and thigh meat samples for female broilers from local Anadolu-T and commercial broiler genotypes.

**Table 5:** Sensory analysis of breast and leg meat of different broiler genotypes (mean ± SEM).

Genotype	Color	Tenderness-	Odor intensity	Moisture	Flavor intensity/	Texture	Aftertaste
		toughness			Taste		
Breast meat							
Anadolu-T	5.45±0.51	3.18±0.73	4.54±0.60	3.27±0.53	4.09±0.70	5.18±0.68	4.27±0.70
Cobb 500	5.45±0.80	3.63±0.70	4.81±0.77	3.36±0.64	4.63±0.65	5.27±0.85	4.18±0.73
Ross 308	4.90±0.51	3.27±0.71	5.00±0.85	3.10±0.65	4.09±0.78	4.54±0.80	3.72±0.78
P value	0.699	0.893	0.903	0.949	0.824	0.772	0.854
Leg meat							
Anadolu-T	5.91±1.38	7.18±1.60	6.36±2.37	6.09±2.59	6.63±2.16	5.36±2.42	6.73±2.10
Cobb 500	6.18±1.60	6.63±1.63	7.09±1.70	5.91±2.02	7.27±1.10	6.09±2.30	7.55±1.37
Ross 308	6.27±1.84	6.00±2.60	6.82±1.77	5.82±2.68	6.61±1.87	5.36±2.11	6.73±1.85
P value	0.861	0.388	0.687	0.965	0.306	0.692	0.477

## 4 Discussion

This study investigated the physico-chemical traits and sensory analysis of female broiler meat of local Anadolu-T and two commercial strains. Physico-chemical meat quality traits include meat color, water-holding capacity, pH, tenderness and nutrient composition, and these attributes generally influence the meat's appearance, taste, shelf life, nutritional value, processing suitability and consumer purchasing decision (Grzybowska-Brzezińska et al., 2023). The shear force test has been the most widely used instrumental approach for measuring meat tenderness (Mudalal et al., 2015), and it might vary among different genetic strains (Maynard et al., 2023, Yaranoğlu et al., 2023) and according to individual and sex (Hailemariam et al., 2022). In this study, as expected, the peak shear force and total shear energy values of breast meat were found to be significantly greater with increasing slaughter age (Li et al., 2019; Rajčić et al., 2024). Peak shear force and total shear energy reveal similar information on the effects of slaughter age on meat texture. In general, the relationship between peak shear force and the other two shear measurements (peak count and shear energy) is highly variable, and this is dependent on the method and meat storage. It is an important parameter in determining meat quality, reflecting the strength and chewiness of muscle fibers (Lu et al., 2024). In our study, there were no significant differences in the shear values of breast meat samples obtained from the local broiler genotype Anadolu-T and commercial chickens. Along with the genotype, shear values can be influenced more by slaughter weight, but inconsistent differences might be observed between high and standard breast-yielding broilers (Mueller et al., 2023). In general, woody breast meat had greater peak shear force and total energy values than normal breast meat. Increased connective tissue accumulation on the surface of woody breast fillets generally needs more compression. If comparing with raw meat, cooked meat had a greater values of peak shear force, shear energy and number of peaks.

In this study, broiler age had a significant effect on almost all breast meat color characteristics, except the hue ( h ) and ΔE values. In breast meat samples, lightness ( $L^{*}$ ), yellowness ( $b^{*}$ ) and chroma ( $C^{*}$ ) values were significantly affected due to genotype differences ( P=0.018 , P=0.015 and P=0.003 ). The breast meat of Cobb 500 had significantly greater yellowness ( $b^{*}$ ) and chrome ( $C^{*}$ ) values, whereas the breast meat of Anadolu-T had significantly greater lightness ( $L^{*}$ ), and significantly lower yellowness ( $b^{*}$ ) and chrome ( $C^{*}$ ) values. Similar to our findings, Yaranoğlu et al. (2023) showed that the lightness ( $L^{*}$ ) value was higher in Anadolu-T compared to Ross 308 and Cobb 500. According to the reference value for meat lightness, all breast meat from all ages was classified as dark meat (Lee et al., 2022). In general, older chickens often presented darker/redder breast meat, and lightness of the leg meat exhibited a decreasing trend with slaughter age (Park et al., 2021). There is a linear relationship between the redness and the pH value of meat; increased pH means the meat color becomes darker (Fletcher, 1999). Meat color is a visual indicator of meat quality for consumers: greater hue values reflects the meat's freshness (Wideman et al., 2016) and is often associated with superior nutritional value and desirability (Harford et al., 2014). The interaction between slaughter age and genotype for color intensity ( $C^{*}$ ) indicated that the breast meat of genotype Anadolu-T had significantly lower $C^{*}$ values than those of genotypes Ross 308 and Cobb 500 at 37 d of age. In contrast, genotype Cobb 500 exhibited significantly higher $C^{*}$ values than the other genotypes at 44 d of age.

Protein is one of the most essential components of the muscle tissues, influencing its nutritional properties and overall quality of broiler meat. Breast meat has slightly higher protein content than leg meat: 22 %–24 % vs. 18 %–21 % (Thanatsang et al., 2020; Özbek et al., 2023). In this study, there were significant differences of moisture and protein levels of chicken breast meat among the genotypes slaughtered at 37 and 44 d of age (DOA). The moisture values of breast meat were significantly increased with slaughter age, whereas crude protein value significantly decreased with age. Moisture is a crucial attribute affecting meat quality, as excessive water loss results in dry and unpalatable meat. In general, the protein content of the breast meat exhibited an increasing trend with increasing slaughter age as muscle mass develops, while moisture content decreases (Park et al., 2021). The opposite finding in this study is likely due to the rapid growth of chicks that can sometimes dilute protein content as a result of increased fat and water accumulation. The breast meat of Cobb 500 showed significantly greater protein value and lower humidity than that of the Anadolu-T and Ross 308 chicken genotypes. A significant slaughter-age–genotype interaction was also found for humidity and protein levels of breast meat, which revealed that the breast meat of Cobb 500 slaughtered at 37 DOA had significantly lower humidity and greater protein level than the other genotypes. Meanwhile, there were no significant differences at 44 DOA among the genotype groups for these traits. Different broiler strains had a varied level of breast muscle growth rate, and this might influence protein deposition in them. Water retention also influences measured protein percentage. The findings of this study support this observation, indicating that increasing slaughter age is associated with humidity and lower protein content in the meat. Breast samples with extremely severe woody breast samples can show a slight decrease in protein levels compared with non-woody breast meat (Madruga et al., 2019). In the main flock where meat quality characteristics were examined, the incidence of breast meat myopathy increased with age, so this may be one reason for the decrease in protein levels with age (Avcı-Küpeli et al., 2025).

The lipid content of raw poultry meat varied between 1.3 % and 6 %, and breast meat fat content was generally under 3 % (Mir et al., 2017). In our study, we found that the fat content of breast meat was the lowest at 1.13 % and the highest at 2.17 %, including all experimental groups. Genotype or slaughter age had no significant effect on the fat content of breast meat. The findings of Akyüz and Onbaşılar (2023) support our finding that genotype had a non-significant effect on fat levels of breast meat. Significant age–genotype interactions for crude fat showed that breast meat samples of Ross 308 slaughtered at 37 DOA and breast meat of Anadolu-T slaughtered at 44 DOA had significantly lower fat levels than those of the others. The fat content influences chicken meat quality, with optimal levels enhancing tenderness. Therefore, controlling fat content during breeding is crucial to ensuring favorable meat quality traits in broiler chickens (Cui et al., 2022).

In this study, slaughter age was not affected by cooking and drip loss of breast meat. Similar to our study, Yaranoğlu et al. (2023) found that there was no significant relationship between gender and genotype in terms of cooking loss and drip loss. However, Li et al. (2019) reported that older chickens often presented lower drip loss in breast meat. The cooking loss value of breast meat of Ross 308 was found to be significantly greater than those of local Anadolu-T and Cobb 500 ( P=0.007 ). In contrast to our study, Hammemi et al. (2024) reported that birds from the Cobb 500 strain exhibited greater drip and cooking losses compared to the Ross 308 genotype. In general, with the increasing slaughter age of broilers, drip loss and cooking loss of breast meat were increased (Rajčić et al., 2024).

In contrast to the findings of Park et al. (2021), the pH of the broiler breast meat exhibited an increasing trend with increasing slaughter age. There were significant differences in breast meat pH values of different genotypes ( P<0.01 ). The breast meat of Cobb 500 had a significantly higher pH value than those of the local Anadolu-T and Ross 308 ( P=0.003 ) broiler chickens. Similar to our result, Yaranoğlu et al. (2023) reported that the pH of fresh breast muscle was found to be higher in the breast meat of the Cobb genotype. In another study, Hammemi et al. (2024) reported that the pH value of meat at 33 d was similar to the Cobb 500 and Ross 308 strains. Significant-age–genotype interactions for meat pH revealed that differences for meat pH were significantly important only at 37 d of age. In contrast, Li et al. (2019) reported that older chickens often have a significantly higher breast muscle pH. The pH value of chicken meat plays a crucial role in determining its flavor, texture and overall quality of meat. The breast meat's ultimate pH is one of the most important traits in describing the technological quality of meat (Beauclercq et al., 2022). The technological quality of meat is a major issue for the competitiveness of commercial broiler meat production as it affects the storage conditions, processing yields, and organoleptic properties of meat and further processed products (Mir et al., 2017). Broiler breast meat is said to have an average pH of 5.7–5.9 under normal conditions. Below 5.7, the meat is referred to as acid; over 6.1, it is dark, firm and dry meat (Riza et al., 2022). For broiler meat, pH values higher than 6.1 or lower than 5.7 are associated with the development of meat quality defects (Baldi et al., 2020; Hammemi et al., 2024). In the present study, the pH levels of the breast meat measured were between 5.72 and 5.96, and it can be said that all breast meat were without defect. The significant interaction between slaughter age and genotype for breast meat pH, moisture, protein and cooking loss revealed that there were significant differences among the genotypes at 37 d of slaughter age, whereas no significant differences were observed among the genotypes at 44 d of slaughter age.

In this study, the breast and thigh meat of chickens from all genotypes showed a similar sensory profile. The sensory attributes of meat from all genotypes were between the hedonic terms “moderate/neutral or slightly under moderate for breast meat” and “slightly greater than control (score 5) for thigh meat”. This finding is important for the consumer in accepting meat derived from local Anadolu-T, newly launched in commercial production. In general, the sensory characteristics of meat vary between sexes and genotypes (Hailemariam et al., 2022), and nutrition has an important impact on most sensory characteristics of meat (Shaviklo et al., 2021; Mogotlane et al., 2024). Muscle fiber properties play a key role in broiler sensory characters because numerous studies have reported the relationships between quality traits and fiber characteristics (Ismail and Joo, 2017). In contrast to our findings, in one study, consumers gave higher sensory scores, except for flavor and off-flavor intensity, of commercial broiler meat than meat from indigenous birds, and the breast meat of the females received higher ratings than that of the males in terms of tenderness, flavor, appearance and general acceptability (Dyubele et al., 2019). In another study, it was reported that breast meat's flavor and odor intensity significantly increased in slatted-floor housing relative to free-range and deep litter (Özbek et al., 2023). The most crucial aspect of poultry meat is its eating quality, and the slaughter age affects the eating and other sensory quality properties of broiler meat. The appearance, texture and flavor intensity are the most appreciated meat quality traits of consumers, and both traits also have a combined effect on the eating quality of broiler meat (Estevéz, 2015; Carvalho et al., 2017).

## 5 Conclusion

Based on the present results, genotype and slaughter age play a crucial role in determining the proximate composition, meat color and sensory characteristics of female broiler breast meat. The local Turkish genotype, *Anadolu-T*, seems to exhibit competitive meat quality compared to commercial broiler strains. In terms of meat quality, our results suggest that local chickens may have potential in commercial production, offering an alternative to the standard commercial strains that dominate the market. In the case of female-only production, the meat quality of broilers was satisfactory in terms of the nutritive values when compared to male broiler chickens and could provide more uniform production instead of mixed-sex broiler production.

## Data Availability

The datasets are available from the corresponding author upon reasonable request.

## References

[bib1.bib1] Akyüz HÇ, Onbaşılar EE (2023). Carcass, visceral organ, and meat quality properties of two broiler hybrids differing in growth rates. Anim Sci J.

[bib1.bib2] Aslan R, Sarıca M, Çavdarcı H, Erensoy K, Karaçay N (2024). The use of partially slatted floor designs as an alternative to littered systems in broiler chickens. I. The effects on the performance, slaughter and carcass traits. Trop Anim Health Pro.

[bib1.bib3] Avcı-Küpeli Z, Küpeli EÇ, Petek M (2025). Histopathologic evaluation of wooden breast and white striping myopathy in different broiler genotypes using light microscopy and image analysis. Rev Científica FCV-LUZ.

[bib1.bib4] Aviagen (2025). ROSS Broiler Pocket Guide.

[bib1.bib5] Baldi G, Yen CN, Daughtry MR, Bodrumer J, Bowker BC, Zhuang H, Petracci M, Gerrard DE (2020). Exploring the factors contributing to the high ultimate pH of broiler pectoralis major muscles affected by wooden breast condition. Front Physiol.

[bib1.bib6] Barbut S, Caballero B, Finglass PM, Toldra F (2016). Encyclopedia of Food and Health.

[bib1.bib7] Beauclercq S, Mignon-Grasteau S, Petit A, Berger Q, Lefèvre A, Métayer-Coustard S, Tesseraud S, Emond P, Berri C, Le Bihan-Duval E (2022). A divergent selection on breast meat ultimate pH, a key factor for chicken meat quality, is associated with different circulating lipid profiles. Front Physiol.

[bib1.bib8] Carvalho RH, Ida EI, Madruga MS, Martinez SL, Shimokomaki M, Estévez M (2017). Underlying connections between the redox system imbalance, protein oxidation and impaired quality traits in pale, soft and exudative (PSE) poultry meat. Food Chem.

[bib1.bib9] Cox R, Kerth CR (2013). Science of Meat Quality.

[bib1.bib10] Cui H, Liu L, Liu X, Wang Y, Luo N, Tan X, Zhu Y, Liu R, Zhao G, Wen J (2022). A selected population study reveals the biochemical mechanism of intramuscular fat deposition in chicken meat. J Anim Sci Biotechno.

[bib1.bib11] Dyubele NL, Muchenje V, Nkukwana TT, Chimonyo M (2019). Consumer sensory characteristics of broiler and indigenous chicken meat: A South African example. Food Qual Prefer.

[bib1.bib12] Ebrahimi M, Daneshyar M, Payvastegan S, Ahmadi H (2026). Association and variation in performance, nutrient digestibility, carcass traits, and intestinal development of broiler chickens by various male-to-female ratios. Vet Anim Sci.

[bib1.bib13] England A, Gharib-Naseri K, Kheravii SK, Wu SB (2023). Influence of sex and rearing method on performance and flock uniformity in broilers – Implications for research settings. Anim Nutr.

[bib1.bib14] Erensoy K, Sarıca M (2023). Fast growing broiler production from genetically different pure lines in Turkey. 2. Broiler traits: Growth, feed intake, feed efficiency, livability, body defects and some heterotic effects. Trop Anim Health Pro.

[bib1.bib15] Estévez M (2015). Oxidative damage to poultry: From farm to fork. Poultry Sci.

[bib1.bib16] Fletcher DL (1999). Broiler breast meat color variation, pH, and texture. Poult Sci.

[bib1.bib17] Grzybowska-Brzezińska M, Banach JK, Grzywińska-Rąpca M (2023). Shaping poultry meat quality attributes in the context of consumer expectations and preferences – A case study of Poland. Foods.

[bib1.bib18] Güler M, Petek M (2026). Effects of on-farm hatching on hatching success and first-week chick performance of local and commercial broiler genotypes. Arch Anim Breed.

[bib1.bib19] Hailemariam A, Esatu W, Abegaz S, Urge M, Assefa G, Dessie T (2022). Effect of genotype and sex on breast meat quality characteristics of different chickens. J Agric Food Res.

[bib1.bib20] Harford ID, Pavlidis HO, Anthony NB (2014). Divergent selection for muscle color in broilers. Poultry Sci.

[bib1.bib21] Hammemi C, Askri A, Létourneau-Montminy MP, Alnahhas N (2024). Current state of breast meat quality in standard-yielding broiler strains. J Appl Poultry Res.

[bib1.bib22] Honikel K O (1998). Reference methods for the assessment of physical characteristics of meat. Meat Sci.

[bib1.bib23] Humane Slaughter Association (HSA) (2013). Practical slaughter of poultry.

[bib1.bib24] IBM Corp (2021). IBM SPSS Statistics 28 Documentation.

[bib1.bib25] Ismail I, Joo ST (2017). Poultry meat quality in relation to muscle growth and muscle fiber characteristics. Korean J Food Sci An.

[bib1.bib26] Keskin M, Şetlek P, Demir S (2017). Renk Ölçüm Sistemlerinin Gıda Bilimleri ve Tarımda Kullanım Alanları. Proceedings of International Advanced Research Engineering Congress.

[bib1.bib27] Kondaiah N, Anjaneyulu ASR, Rao VK, Sharma N, Joshi HB (1985). Effect of salt and phosphate on the quality of buffalo and goat meats. Meat Sci.

[bib1.bib28] King DA, Hunt MC, Barbut S, Claus JR, Cornforth DP, Joseph P, Kim YH, Lindahl G, Mancini RA, Nair MN, Merok KJ, Milkowski A, Mohan A, Pohlman F, Ramanathan R, Raines CR, Seyfert M, Sørheim O, Suman SP, Weber M (2023). American Meat Science Association Guidelines for Meat Color Measurement. Meat and Muscle Biology.

[bib1.bib29] Kralik G, Kralik Z, Grcevic M, Hanzek D, Banu Y (2018). Animal Husbandry and Nutrition.

[bib1.bib30] Li J, Yang C, Peng H, Yin H, Wang Y, Hu Y, Yu C, Jiang X, Du H, Li Q, Liu Y (2019). Effects of slaughter age on muscle characteristics and meat quality traits of Da-Heng meat type birds. Animals.

[bib1.bib31] Löhren U (2012). Overview on current practices of poultry slaughtering and poultry meat inspection. EFSA Support Publ.

[bib1.bib32] Lu T, Abdalla Gibril BA, Xu J, Xiong X (2024). Unraveling the genetic foundations of broiler meat quality: Advancements in research and their impact. Genes.

[bib1.bib33] Madruga MS, da Rocha TC, de Carvalho LM, Sousa AMBL, de Sousa Neto AC, Coutinho DG, de Carvalho Ferreira AS, Soares AJ, de Sousa Galvão M, Ida EI, Estévez M (2019). The impaired quality of chicken affected by the wooden breast myopathy is counteracted in emulsion-type sausages. J Food Sci Technol.

[bib1.bib34] Maynard CJ, Jackson AR, Caldas-Cueva JP, Mauromoustakos A, Kidd MT, Rochell SJ, Owens CM (2023). Meat quality attributes of male and female broilers from 4 commercial strains processed for 2 market programs. Poultry Sci.

[bib1.bib35] Meira M, Afonso IM, Casal S, Lopes JC, Domingues J, Ribeiro V, Dantas R, Casal S, Brito NV (2022). Carcass and meat quality traits of males and females of the “Branca” Portuguese autochthonous chicken breed. Animals.

[bib1.bib36] Mir NA, Rafiq A, Kumar F, Singh V, Shukla V (2017). Determinants of broiler chicken meat quality and factors affecting them: A review. J Food Sci Technol.

[bib1.bib37] Mogotlane PM, Ng'ambi JW, Nyazema NZ, Chitura T (2024). Effect of feed supplementation with probiotics and antimicrobial agents on meat quality of broiler chickens. Agric Sci Dig.

[bib1.bib38] Mueller AJ, Maynard CJ, Jackson AR, Mauromoustakos A, Kidd MT, Rochell SJ, Caldas-Cueva JP, Sun X, Giampietro-Ganeco A, Owens CM (2023). Assessment of meat quality attributes of four commercial broiler strains processed at various market weights. Poultry Sci.

[bib1.bib39] Mudalal S, Lorenzi M, Soglia F, Cavani C, Petracci M (2015). Implications of white striping and wooden breast abnormalities on quality traits of raw and marinated chicken meat. Animal.

[bib1.bib40] Özbek M, Petek M, Ardıçlı S (2020). Physical quality characteristics of breast and leg meat of slow- and fast-growing broilers raised in different housing systems. Arch Anim Breed.

[bib1.bib41] Özbek M, Petek M, Çetin E, Çetin İ (2023). Influence of genotype and housing systems on the incidence of white striping, proximate composition and sensory analysis of broiler breast meat. Acta Vet Eurasia.

[bib1.bib42] Park SY, Byeon DS, Kim GW, Kim HY (2021). Carcass and retail meat cuts quality properties of broiler chicken meat based on the slaughter age. J Anim Sci Technol.

[bib1.bib43] Rajčić A, Bošković Cabrol M, Glišić M, Čobanović N, Tomović V, Laudanović M, Nesic S (2024). Pathomorphological and meat quality alterations connected with wooden breast in broiler chickens of different genotypes and slaughter ages. Acta Vet.

[bib1.bib44] Riza MK, Bintoro VP, Kismiyati S, Sugiharto S (2022). Carcass and meat karakteristiği of broilers stocked at a high density and supplemented with acidified turmeric. Adv Anim Vet Sci.

[bib1.bib45] Samakradhamrongthai RS, Chaiwang N, Jaturasitha S, Arjin C, Sringarm K, Bunmee T, Renaldi G, Buajai T, Setthaya P (2026). Effects of genotype and sex on carcass characteristics, meat quality, volatile compounds, and flavor profiles of Thai native chickens and broilers. Poultry Sci.

[bib1.bib46] Selvi TN, Üstüner H, Çakıroğlu KA, Petek M, Çavuşoğlu E, Demir M, Küpeli EÇ (2026). Evaluation of growth performance, meat quality and carcass characteristics of slow and fast-growing broiler chickens under commercial conditions. J Appl Poultry Res.

[bib1.bib47] Shaviklo AR, Alizadeh-Ghamsari AH, Hosseini SA (2021). Sensory attributes and meat quality of broiler chickens fed with mealworm (Tenebrio molitor). J Food Sci Technol.

[bib1.bib48] Siddique A, Black MT, Alvarado BW, Garner L, Huang TS, Gupta A, Wilson AE, Sawyer JT, Morey A (2024). Effect of age, deboning time of carcass, and different cooking conditions on the woody breast myopathies in chicken: A meta-analysis. Foods.

[bib1.bib49] Snedecor GW, Cochran WG (1989). Statistical Methods.

[bib1.bib50] Starbuck C (2023). The fundamentals of people analytics.

[bib1.bib51] Suliman GM, Hussein EO, Al-Owaimer AN, Qaid MM, Al-Garadi MA, Swelum AA, Alshamiry FA (2026). Growth performance, carcass traits, meat quality, and economic efficiency of ROSS 308 broilers at different slaughter ages and sexes. Cogent Food Agric.

[bib1.bib52] Tagem Arge, Innovasyon (2020). Yerli etlik damızlık tavuk ıslah çalışmaları (in Turkish).

[bib1.bib53] Thanatsang KV, Malila Y, Arayamethakorn S, Srimarut Y, Tatiyaborworntham N, Uengwetwanit T, Panya A, Rungrassamee W, Visessanguan W (2020). Nutritional properties and oxidative indices of broiler breast meat affected by wooden breast abnormality. Animals.

[bib1.bib54] Thiex NWJ, Latimer Jr GW (2023). Official Methods of Analysis of AOAC INTERNATIONAL.

[bib1.bib55] Topal E, Petek M (2021). Effects of fully or partially slatted flooring designs on the performance, welfare and carcass characteristics of broiler chickens. Brit Poultry Sci.

[bib1.bib56] Tura M, Gagliano MA, Valli E, Petracci M, Toschi TG (2024). A methodological review in sensory analyses of chicken meat: Sensory analyses of chicken meat: A review. Poultry Sci.

[bib1.bib57] Turkish Official Gazette (TC Resmi gazete) (2011). Regulation on specific hygiene rules for foods of animal origin (Hayvansal Gıdalar için özel hijyen kuralları yönetmeliği), No: 28155-27.12.2011.

[bib1.bib58] Wichchukit S, O'Mahony M (2015). The 9-point hedonic scale and hedonic ranking in food science: some reappraisals and alternatives. J Sci Food Agr.

[bib1.bib59] Wideman N, O'bryan CA, Crandall PG (2016). Factors affecting poultry meat colour and consumer preferences – A review. World Poultry Sci J.

[bib1.bib60] Wheeler TL, Shackelford SD, Johnson LP, Miller MF, Miller RK, Koohmaraie M (1997). A comparison of Warner-Bratzler shear force assessment within and among institutions. J Anim Sci.

[bib1.bib61] Yaranoğlu B, Eyi R, Yaranoglu MH (2023). Comparative investigation of Ross and Cobb broiler genotypes with different gender in terms of slaughter, carcass characteristics and some meat quality traits. Spec J Agric Sci.

[bib1.bib62] Yue K, Cao QQ, Shaukat A, Zhang C, Huang SC (2024). Insights into the evaluation, influential factors and improvement strategies for poultry meat quality: A review. NPJ Sci Food.

[bib1.bib63] Zhuang H, Savage EM (2013). Comparison of cook loss, shear force, and sensory descriptive profiles of boneless skinless white meat cooked from a frozen or thawed state. Poult Sci.

